# Zerovalent Fe, Co and Ni nanoparticle toxicity evaluated on SKOV‐3 and U87 cell lines

**DOI:** 10.1002/jat.3220

**Published:** 2015-09-17

**Authors:** Rosalba Gornati, Elisa Pedretti, Federica Rossi, Francesca Cappellini, Michela Zanella, Iolanda Olivato, Enrico Sabbioni, Giovanni Bernardini

**Affiliations:** ^1^ Department of Biotechnology and Life SciencesUniversity of InsubriaVareseItaly; ^2^ VenetonanotechECSINRovigoItaly; ^3^ Interuniversity Center ‘The Protein Factory’, Politecnico di MilanoICRM‐CNR Milano and Università dell'InsubriaMilanItaly

**Keywords:** Nanoparticles, dissolution, cytotoxicity, gene expression, uptake

## Abstract

We have considered nanoparticles (NPs) of Fe, Co and Ni, three transition metals sharing similar chemical properties. NP dissolution, conducted by radioactive tracer method and inductively coupled plasma mass spectrometry, indicated that NiNPs and FeNPs released in the medium a much smaller amount of ions than that released by Co NPs. The two considered methodological approaches, however, gave comparable but not identical results. All NPs are readily internalized by the cells, but their quantity inside the cells is less than 5%. Cytotoxicity and gene expression experiments were performed on SKOV‐3 and U87 cells. In both cell lines, CoNPs and NiNPs were definitely more toxic than FeNPs. Real‐time polymerase chain reaction experiments aimed to evaluate modifications of the expression of genes involved in the cellular stress response (HSP70, MT2A), or susceptible to metal exposure (SDHB1 and MLL), or involved in specific cellular processes (caspase3, IQSEC1 and VMP1), gave different response patterns in the two cell lines. HSP70, for example, was highly upregulated by CoNPs and NiNPs, but only in SKOV‐3 cell lines. Overall, this work underlines the difficulties in predicting NP toxicological properties based only on their chemical characteristics. We, consequently, think that, at this stage of our knowledge, biological effects induced by metal‐based NPs should be examined on a case‐by‐case basis following studies on different *in vitro* models. Moreover, with the only exception of U87 exposed to Ni, our results suggest that metallic NPs have caused, on gene expression, similar effects to those caused by their corresponding ions. Copyright © 2015 The Authors. Journal of Applied Toxicology published by John Wiley & Sons, Ltd.

## Abstract 2

We have considered Fe, Co and Ni nanoparticles and studied their dissolution by radioactive tracer method and ICP‐MS. We have also performed cytotoxicity and gene expression experiments on two different cell lines. The obtained results convinced us that, at this stage of our knowledge, metal‐based nanoparticles should be examined on a case‐by‐case basis on different in vitro models. Moreover, our results suggest that metal‐based nanoparticles have caused similar effects to those caused by their corresponding ions.

## Introduction

Nanosciences and nanotechnologies are seen as having a huge potential to bring benefits to many areas of science, technology and everyday life. At the same time, it is recognized that their application may raise new challenges in the safety, regulatory or ethical domains that must be assessed (Schug *et al*., 2014b). Adequately assessing the biological effects of nanoparticles (NPs) is particularly difficult as their *in vitro* and *in vivo* behaviours depend on numerous factors, including composition, size, shape, route of exposure or dosage (Di Gioacchino *et al*., 2009; Gornati *et al*., 2009). Additionally, unforeseen interferences affecting the outcome of a particular toxicological assay, inadequate controls, the wide range of biological assays, the different methods of quantifying dose and aggregation are factors that contribute to confound the issue. Quite possibly for these reasons, some of the results obtained are conflicting (Choi *et al*., 2013). Moreover, metal ions released from their NPs (dissolution) also need to be taken into account; there are indeed recent studies showing that particles can gradually dissolve in the culture medium generating ions and that toxicity could be due to the released ions (Papis *et al*., 2007b; Sabbioni *et al*., 2014a; Semisch *et al*., 2013; Stebounova *et al*., 2012).

Here, we have considered zerovalent NPs of Co, Fe and Ni, three transition metals of the same group (group VIIIb) of the periodic table. CoNPs are promising for nanotechnological applications such as pigments, catalysis, sensors, electrochemistry, magnetism, energy storage and magnetic fluids (Bedanta *et al*., 2013; Liu *et al*., 2005) as well as nanomedicine where it finds applications as highly sensitive magnetic resonance imaging contrast agents (Bouchard *et al*., 2009; Lacroix *et al*., 2013; Parkes *et al*., 2009). Zerovalent FeNPs have received attention for groundwater remediation. Still, there are many concerns on their fate and transport and the corresponding risks (Cundy *et al*., 2008; Gottschalk and Nowack, 2011; Zhu *et al*., 2011). NiNPs embedded in a mesoporous silica material have excellent potential for catalytic applications, while films of NiNPs are used as high‐frequency field‐amplifying components (Morozov *et al*., 2011; Wessells *et al*., 2006). Ni, however, represents a well‐known problem for allergy and dermatitis (Braga *et al*., 2013).

In this paper, we have faced aspects of dissolution and cellular uptake of zerovalent metallic NPs as well as gene expression modifications they can cause. For metal or metal oxide NPs, dissolution is an important characteristic (Benetti *et al*., 2014) and has to be taken into consideration, in particular when it occurs after internalization (Jiang *et al*., 2012). Indeed, the intracellularly released ions (‘Trojan horse effect’) may account for the observed cytotoxicity (Ortega *et al*., 2013). The evaluation of NP uptake is also important, but cytosolic levels of metal ions are more difficult to assess than those of culture media (Sabbioni *et al*., 2007) and therefore have been largely ignored. We have tried to fill this gap determining the amount of NPs internalized by the cells. Furthermore, we have studied the effect of Fe‐, Co‐ and NiNPs and corresponding ions on the expression of a selected panel of genes known to be susceptible to metal exposure or involved in a cellular stress response and in other specific cellular processes.

## Materials and methods

### Metal nanoparticles

Zerovalent metal NPs were purchased from American Elements (Los Angeles, CA, USA) with the following characteristics: FeNPs (purity 99.9%, aerodynamic particle size < 100 nm), NiNPs (purity, 99.9% aerodynamic particle size < 100 nm) and CoNPs (purity 99.5% aerodynamic particle size < 100 nm).

NPs were checked by transmission electron microscopy (TEM) using a JEOL‐1010 electron microscope (Tokyo, Japan) equipped with a CCD camera MORADA (Olympus, Tokyo, Japan) operating at 90 kV. Samples for TEM were prepared placing 5 µl of a diluted suspension of each NP in ethanol on a formvar carbon‐coated copper grid (Bava *et al*., 2013a).

### Dissolution experiments by radiotracing method

Radiolabelled FeNPs (^59^FeNP, T_1/2_ = 44.5 days, specific radioactivity 434.4 kBq (1.2 μCi)/mg Fe), CoNPs (^60^CoNP, T_1/2_ = 5.2 years, specific radioactivity 9.25 MBq (0.25 mCi)/mg Co) and (^58^Co)‐NiNPs (^58^Co‐NiNP, T_1/2_ = 71.3 days, specific radioactivity 91.7 kBq (2.48 μCi)/mg Ni) were prepared by 12 or 96 h irradiation of 10 mg of dry Fe, Co and NiNPs in the thermal neutron flux of 2 × 10^14^ neutrons cm^–2^ s^–1^ of the nuclear reactor HFR (Petten, The Netherlands). From ^58^Ni, the epithermal neutron activation process produces ^58^Co. This radioactive species has been successfully used as a tracer of Ni in rat tissues (Kalliomäki *et al*., 1986).

The irradiated NPs were suspended in 10 ml MilliQ H_2_O and centrifuged at 25 000 *g* for 10 min to eliminate traces of soluble ^59^Fe, ^60^Co and ^58^Co species generated from raw NP impurities or by recoil atom reactions during the irradiation.

A portion of radiolabelled NPs was then submitted for a stability test to assess whether the ^59^Fe, ^60^Co and ^58^Co were firmly bound to the NPs, and therefore suitable as NP radiomarkers in biological media. For the stability test, ^59^FeNP, ^60^CoNP and ^58^Co(Ni)NP were resuspended in 2 ml MilliQ H_2_O to a final concentration of 100 μm, dispersed by an ultrasonic bath for 15 s, stirred for 30 min and ultracentrifuged for 30 min at 60 000 *g* at 4 °C. The ^59^Fe, ^60^Co and ^58^Co were measured in the supernatants. As a result, only a tiny fraction of < 0.8% of the three radiotracers was recovered in the supernatants indicating that ^59^Fe, ^60^Co and ^58^Co(Ni) radiolabels in the aqueous environment were firmly bound to the NPs and that the radioactive particles were suitable for the dissolution test.

To evaluate the dissolution, the radiolabelled NPs were resuspended in 10 ml RPMI culture medium to a final concentration of 100 μm and incubated for 4 or 72 h at 37 °C, 5% CO_2_ and 95% humidity. After centrifugation at 25 000 *g* for 10 min, ^59^Fe, ^60^Co and ^58^Co in the supernatants were counted by the automatic integral γ‐counting system (Wallac 1480 3′′, Wallac, Sweden) equipped with a well type NaI (Tl) detector, using the windows of 950–1400 KeV (^59^Fe), 800–2000 KeV (^60^Co) and 600–1800 KeV (^58^Co). Experiments were performed in triplicate.

### Dissolution experiments by inductively coupled plasma mass spectrometry

Ten millilitres of a 4 mm NP suspension, sonicated for 15 min in a water bath, were diluted to a final concentration of 100 μm in RPMI‐1640 supplemented with 10% fetal bovine serum (FBS). Fifteen millilitres of each sample were placed in 10 cm Petri dishes and kept at 37 °C for 0 and 72 h in the presence of 5% CO_2_. After incubation, samples were centrifuged four times at 8000 *g* for 5 min and ultracentrifuged at 300 000 *g* for 2 h at 4 °C to remove undissolved NPs. The supernatants were filtered using a 0.22 µm pore size membrane (Millipore, Milan, Italy).

The analysis was performed, after a 1: 10 dilution of the samples with 2% ultrapure HNO_3_, by inductively coupled plasma mass spectrometry (ICP‐MS, NexION 300D; Perkin‐Elmer, Billerica, MA, USA). The calibration curve was performed by the multi‐element calibration standard 3 method (Perkin‐Elmer) using a range of concentration of 10–700 ppb (µg l^–1^) for each element. The results were analysed by the NexION Instrument Control Session (Perkin‐Elmer).

### Cell culture

SKOV‐3 (human ovarian carcinoma) cell line were maintained as adherent cells at 37 °C and a humidified 5% CO_2_ atmosphere in RPMI‐1640 medium supplemented with 10% FBS, 1% l‐glutamine and 1% penicillin/streptomycin solution. The U87 (human glioblastoma) cell line was maintained, at the same condition, in DMEM medium supplemented with 10% FBS, 1% l‐glutamine, 1% sodium pyruvate and 1% penicillin/streptomycin solution. Cell lines, cell culture media and supplements were purchased from Sigma (Milan, Italy); plastics were purchased by Euroclone SpA (Milan, Italy).

### Cell viability

Cell viability was determined measuring ATP content by the CellTiter‐Glo Assay (Promega, Milan, Italy) according to the manufacturer's instructions. Briefly, 200 µl of cell suspension (containing 2 × 10^4^, 1 × 10^4^, 5 × 10^3^ or 2.5 × 10^3^ cells, depending on the exposure time) were seeded into 96‐well assay plates and cultivated for 24 h at 37 °C in 5% of CO_2_ to equilibrate and become attached before treatment. Cells were then exposed to 100 μL of increasing concentrations (0–160 µg) of CoNPs, FeNPs or NiNPs for 0.5, 1, 2, 24, 48 and 72 h. NP suspensions used in these experiments were freshly prepared each time, sonicated for 15 min and diluted according to the experimental protocol. The range of concentrations has been determined for each NP in preliminary experiments (range finding test) expressly run for this purpose. After the treatment, plates were equilibrated for 30 min at room temperature and then 100 µl of CellTiter‐Glo reagent was added to each well. Plates were shaken for 2 min and left at room temperature for 10 min before recording luminescent signals using the Infinite F200 plate reader (Tecan Group, Männedorf, Switzerland). Each plotted value is the mean of three different experiments.

### Cellular localization and uptake quantification

For these experiments, performed in steady state, 10^6^ cells were seeded in a 10 cm Petri dish, cultivated at 37 °C in 5% CO_2_ to reach a 70–80% confluence before treatment, and then exposed, for 30 min and 3 h, to 10 µg 100 µl^–1^ of CoNPs, or NiNPs, or 5 µg 100 µl^–1^ of FeNP. For TEM analysis, cells were harvested, fixed and embedded in an Epon‐Araldite as previously described (Bava *et al*., 2013b).

Quantification of the internalized metals was evaluated by ICP‐MS of mineralized cells. Therefore, cells exposed to NPs for 30 min were washed twice with PBS, collected in a Teflon tube and dried in a microwave oven at 105 °C. Twelve millilitres of ultrapure HNO_3_/HCl (3: 1 v/v) were added to the samples then placed in a microwave MARS5 express (CEM, Bergamo, Italy) at the following conditions: 1600 W, the temperature was raised to 175 °C ± 5 °C at 30 °C min^–1^ and maintained at 175 °C for 4 min and 30 s. Measurements were performed by NexION 300D ICPMS apparatus (Perkin‐Elmer) after 1/100 dilution of the samples with 2% ultrapure HNO_3_ as reported in the Dissolution experiments by inductively coupled plasma mass spectrometry.

### RNA extraction, retrotranscription and qualitative polymerase chain reaction

About 2 × 10^6^ SKOV‐3 and U87 cells were exposed at 5 µg 100 µl^–1^ (about 1 mm concentration that causes a 20–30% of ATP reduction) of CoNPs, FeNPs or NiNPs for 24 h. Exposed and not exposed cells were then washed twice with PBS and harvested. We have also arranged a set of samples in which cells were exposed, for the same time, to CoCl_2_.6H_2_O, FeCl_3_ or NiCl_2_ at a nominal concentration of 5 µg 100 µl^–1^ in terms of Co, Fe and Ni. Total RNA was extracted by the TRIzol method according to the manufacturer's instructions. The extracted RNA was quantified by spectrophotometer, its integrity was checked by gel electrophoresis and then it was retrotranscribed as previously reported (Gornati *et al*., 2004). The generated cDNA was stored at –20 °C.

Polymerase chain reaction (PCR) was performed using 1 µl of cDNA and 1 μm solution of specific primers (BMR Genomics, Padova, Italy) designed within coding sequence of selected genes, cytoplasmatic β‐actin was used as a housekeeping gene. For each sample, a set of PCR has been run without retrotranscription (Tognoli *et al*., 2011).

### Retrotranscription and real‐time polymerase chain reaction

The first strand cDNA was synthesized using the High Capacity cDNA Reverse Transcription Kit (Life Technologies, Monza, Italy) according to the manufacturer's instructions. The real‐time PCR was performed with specific TaqMan probes (Life Technologies) designed within the sequences of a panel of genes selected on the basis of the results of the qualitative PCR [β‐actin, Hs03023880_g1; vacuole membrane protein 1 (VMP1), Hs00229548_m1; caspase 3 (CASP3), Hs00234387_m1; heat shock protein 70 (HSP70), Hs00399163_s1; IQ motif and sec7 domain 1 (IQSEC1), Hs00208333_m1; mixed lineage leukaemia (MLL), Hs00610538_m1; metallothionein IIA (MT2A), Hs01591333_g1; succinate dehydrogenase, iron sulphur subunit 1 (SDHB1), Hs01042478_g1]. Each reaction tube was set up according to Papis *et al*. (2007a), split in two wells and run in the ABI Prism 7000 Sequence Detection System thermocycler (Applied Biosystems, Monza, Italy). The experiment was repeated three times.

### Statistical analysis

The Ct values were recorded and the relative gene expression, expressed as 2^–ΔΔ*C*t^ (ΔCt = Ct_Target_ – Ct_β‐actin_ and ΔΔCt = ΔCt_Exposed samples_ – ΔCt_Control samples_), was taken as dependent variables. Data analysis was performed by one‐way ANOVA analysis (α = 0.05), completed with Scheffé's test (*P* < 0.05) to determine which groups (NPs, ions) were significantly different from control. Standard errors of real‐time experiments were computed by taking into account propagation of errors (Nordgård *et al*., 2006).

## Results

### Nanoparticle characterization and dissolution experiments

**Figure 1:**
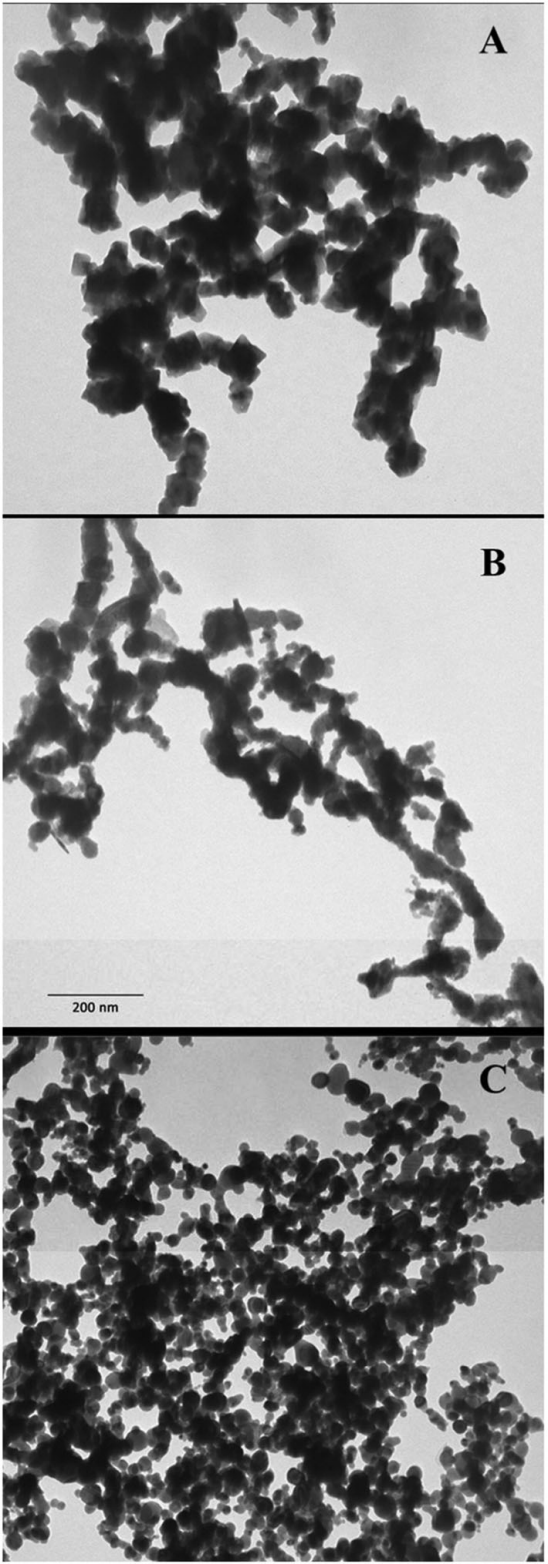
Transmission electron microscopy images of metal nanoparticles. Fe (A), Co (B) and Ni (C) nanoparticles were deposited on formvar carbon‐coated grids and observed at the same enlargement. Certain heterogeneity in the size and shape is observed for all the nanoparticles. The 200 µm scale bar reported in (B) is referred to the entire figure.

The NPs used in this work were analysed by TEM. As shown in Fig. 1(A–C), the dry powder displayed different shapes and sizes (10–50 nm).

**Figure 2:**
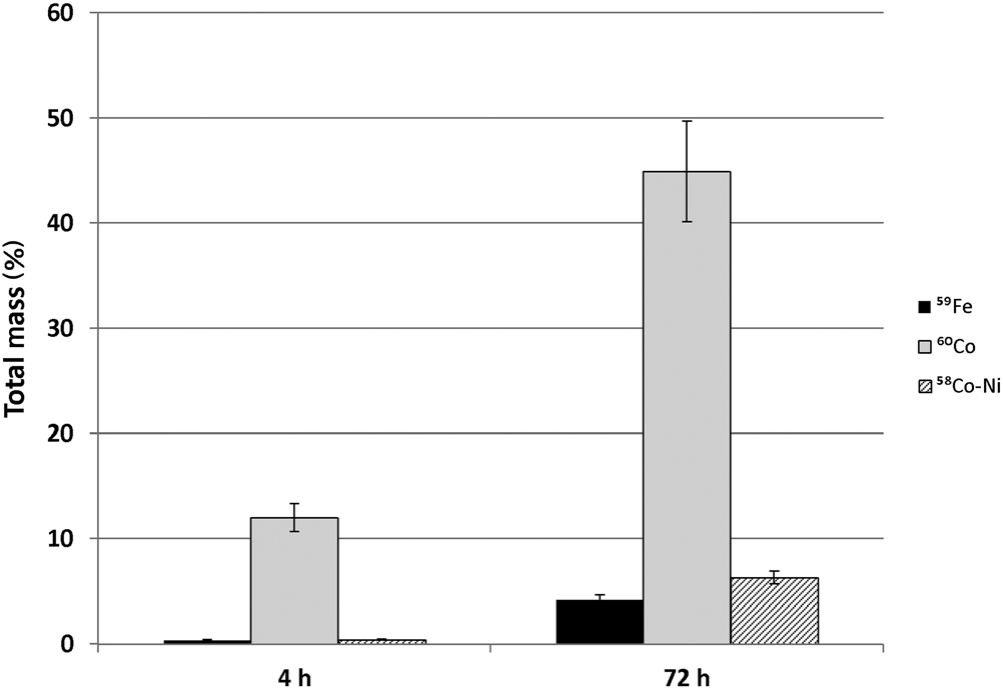
Dissolution at time 4 and 72 h, in RPMI‐1640 culture medium, of ^59^Fe, ^60^Co and ^58^Co‐Ni nanoparticles. Results are expressed as percentage of the initial mass of nanoparticles. Bars represent standard errors.

The dissolution of Co‐, Fe‐ and NiNPs is expressed as a percentage of ions released from the working suspension (Fig. 2). Already after 4 h in culture medium (37 °C and 5% CO_2_), CoNPs released a significant amount of metal (about 12% of the total mass) and this percentage increased to 44% after 72 h of incubation. NiNPs and FeNPs, instead, released a smaller amount of ions (6% and 4% of their mass). Similar behaviour was observed in experiments in which measurements were performed by ICP‐MS (data not shown).

### Cell viability

**Figure 3:**
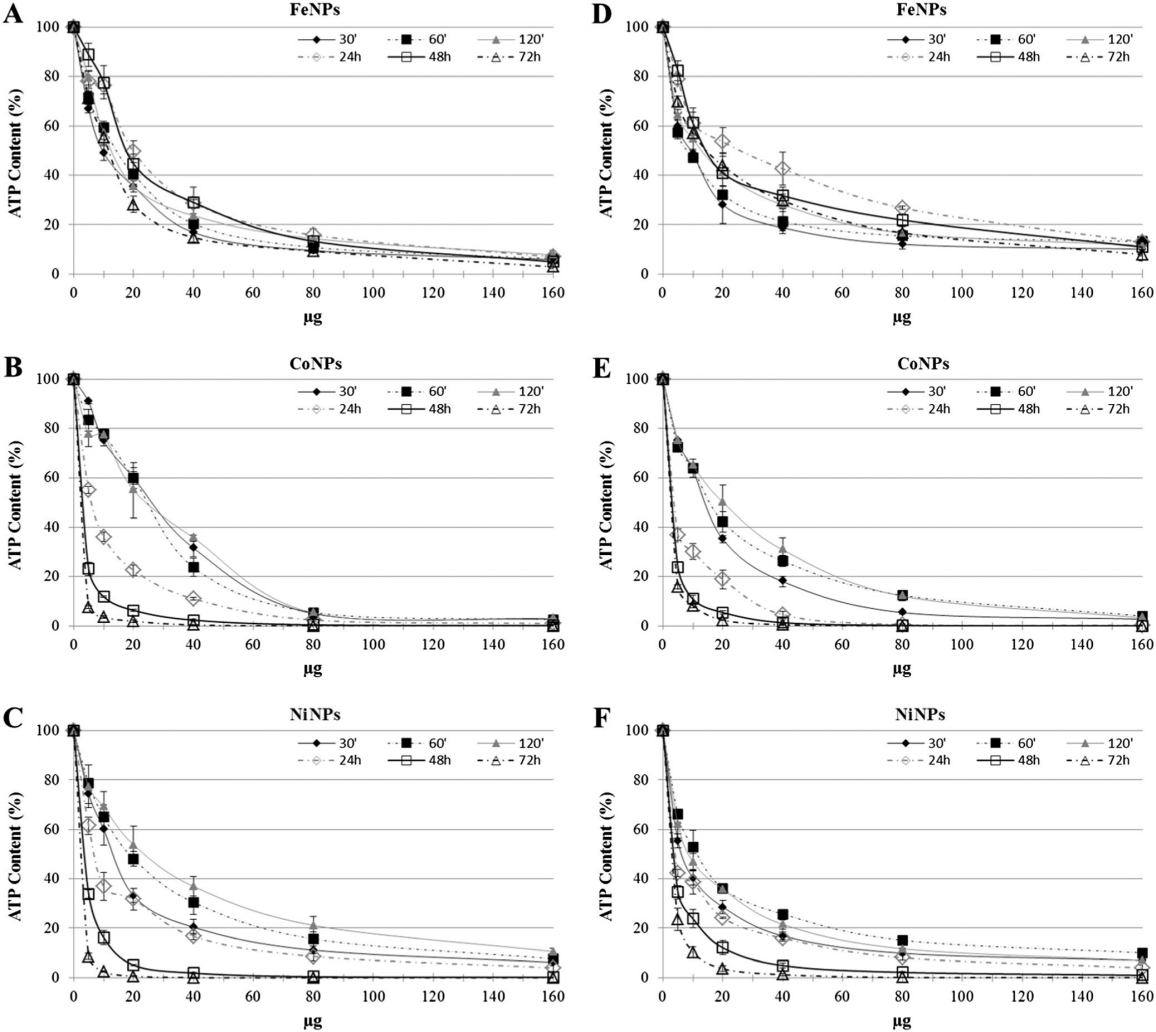
Percentage of ATP content, normalized to control, in SKOV‐3 and in U87 exposed to FeNPs (A,D), CoNPs (B,E) and NiNPs (C,F) for different times. Bars represent standard errors. ATP, adenosine triphosphate; NPs, nanoparticles.

Co‐, Fe‐ and NiNP cytotoxicity on SKOV‐3 and U87 cells was evaluated by measuring cellular ATP content after exposure for 30 min, 60 min, 120 min, 24 h, 48 h and 72 h in culture medium. As shown in Fig. 3(A–F), a dose‐dependent reduction in cell viability was observed for all the NPs; moreover, both CoNPs and NiNPs had also elicited a time‐dependent response. As expected, the results of cell viability for the three NPs were significantly different, in particular, at low concentrations and long‐term exposure. Cytotoxicity on SKOV‐3 and U87 was ranked as follows: CoNPs > NiNPs > FeNPs. Apparently, we have not observed a significantly different sensitivity between the two cell lines, and, at very low concentrations (1–10 µg 100 µl^–1^), we have not been able to observe hormetic effects (Calabrese, 2008) for any tested NP.

### Cellular localization and uptake quantification

**Figure 4:**
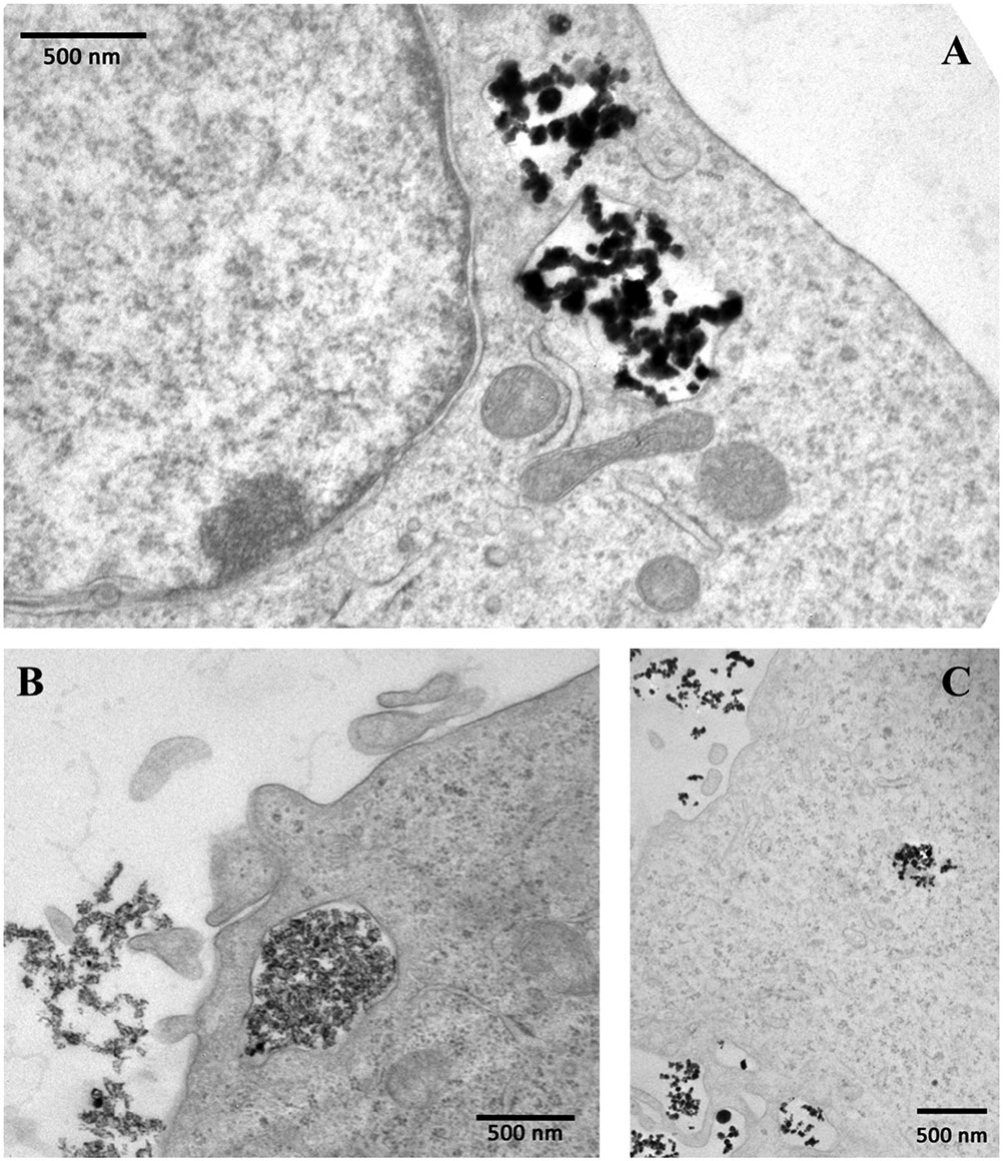
Transmission electron microscopy images of SKOV‐3 cells exposed to FeNPs (A), CoNPs (B) and NiNPs (C) for 30 min. NPs, when inside the cell are localized inside vesicles. There are no free NPs in the cytoplasm. Nucleus and mitochondria do not contain NPs. NPs, nanoparticles.

TEM images of SKOV‐3 cells exposed, reported as examples in Fig. 4(A–C), confirmed that all NPs were promptly internalized. After 30 min of exposure, all the NPs appeared inside the cells, suggesting that the internalization was aspecific, and not time dependent. In these images, NPs are identified as high electron density objects as NPs sufficiently maintained the morphology observed in the cell‐free environment (Fig. 1). Cellular pseudopodes, characteristic structures of the endocytosis pathway, are particularly evident even though we cannot exclude that the internalization, at least for the smallest NPs, could occur by direct crossing of the plasma membrane lipid bilayer. Once entered, most of the NPs remained in the cytoplasm inside the vesicles. It is worth noting that, also after 3 h of exposure, no NPs were observed in the nuclei even though a massive internalization of NPs can modify the nuclear shape as already reported in a previous paper (Bava *et al*., 2013a).

**Figure 5:**
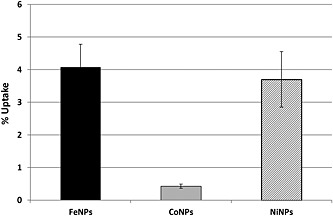
Quantitative evaluation of the cellular uptake of Fe, Co and Co‐NiNPs expressed as percentage of the initial mass of NPs. Bars represent standard errors. NPs, nanoparticles.

Quantitative evaluation of the cellular uptake of NPs via determination of the total content of Fe, Co and Ni was approximately 4% for FeNPs and NiNPs and 0.4% for CoNPs (Fig. 5). These low values could be due to a tendency of CoNPs to aggregate, to their high solubility and to the experimental conditions.

### Gene expression

A panel of genes to be tested has been identified based on our previous experience and published data (Papis *et al*., 2014). After the preliminary results obtained by qualitative PCR, we have quantitatively evaluated the expression of the most responsive genes of this panel (i.e,, β‐actin; VMP1; CASP3; HSP70; IQSEC1; MLL; MT2A; SDHB1) in the two cell lines exposed for 24 h to Fe‐, Co‐ and NiNPs and their corresponding ions. β‐actin was used as reference gene.

First, we checked that the concentration used for CoCl_2_, FeCl_3_ and NiCl_2_ exposure was not highly toxic. A cell viability test has confirmed that the decrease of ATP content was in the 10–40% range (data not shown). The genes considered in this study are those generally involved in cellular stress response, such as HSP70 and MT2A, or susceptible to metal exposure, such as SDHB1 and MLL, or those more closely involved in specific cellular processes, such as CASP3, IQSEC1 and VMP1.

**Figure 6:**
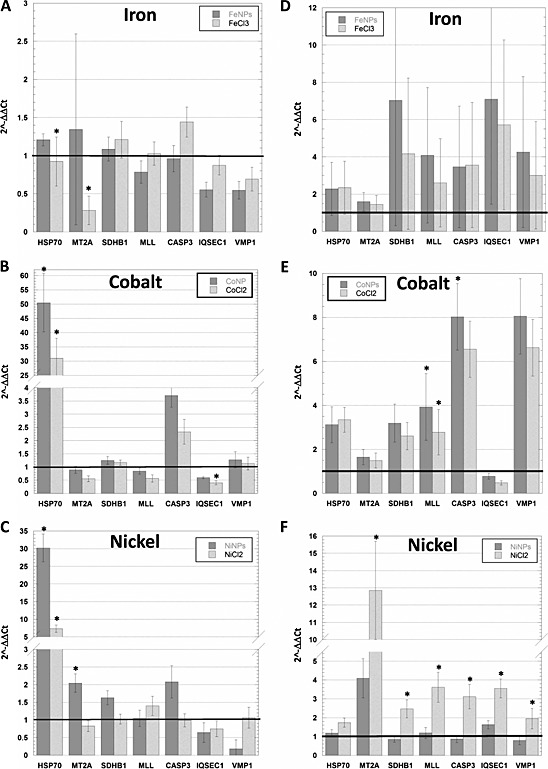
Real‐time polymerase chain reaction of HSP70, MT2A, SDHB1, MLL, CASP3, IQSEC1 and VMP1 in control and exposed cells (5 µg 100 µl^–1^ of Fe‐, Co‐ or NiNPs) normalized with the reference gene β‐actin. (A–C) SKOV‐3; (D–F) U87. The gene expression is reported as fold change compared to unexposed cells (black line). *Scheffé's test, *P* < 0.05. CASP3, caspase 3; HSP70, heat shock protein 70; IQSEC1, IQ motif and sec7 domain 1; MLL, mixed lineage leukaemia; MT2A, metallothionein; NPs, nanoparticles; SDHB1, iron sulphur subunit 1; VMP1, vacuole membrane protein 1.

We have observed that the two cell lines exhibited a different behaviour to treatments. In particular, U87 seemed to be more responsive than SKOV‐3. FeNPs and FeCl_3_ did not seem to affect too much gene expression in SKOV‐3 with the exception of a downregulation of HSP70 and MT2A, as highlighted in Fig. 6(A). CoNPs and ions were more effective in inducing a significant increase of HSP70 expression; furthermore, Co ions also caused a reduction of IQSEC1 (Fig. 6B). As for Co, NiNPs and ions also caused an overexpression of HSP70, while MT2A expression is induced by NiNPs, but not by Ni^2+^ (Fig. 6C). Figure 6(D–F) reports a gene expression profile in U87. In this case, both FeNPs and ions had no influence on the expression of all considered genes (Fig. 6D). However, as shown in Fig. 6(E), CoNPs and CoCl_3_ have induced an overexpression of MLL, and CoNPs increased CASP3 mRNA. Concerning Ni (Fig. 6F), unexpectedly, the NP form has not influenced the expression of all the considered genes; conversely, Ni^2+^ has raised the level of all gene panels except for HSP70.

Data analysis of the relative gene expression, completed with Scheffé's test when *P* < 0.05, showed that in SKOV‐3 while HSP70 was significantly modified by almost all treatments (Fig. 6A–C), few other genes were sensitive to the treatment; specifically, MT2A has been downregulated by Fe ions and upregulated by NiNPs, while IQSEC1 has been downregulated by Co ions. For what concern U87 cell line, FeNPs and ions did not cause any significant change in all the examined genes. After exposure of the cells with CoNPs, U87 showed the upregulation of MLL and CASP3, whereas Co ions influence only the expression of MLL. Ni ions but not NPs have affected all the genes with the exception of HSP70 (Fig. 6D–F). As shown in the histograms in Fig. 6, it would appear that most of these genes are affected by treatment. This can happen when the ΔCt standard deviation, a measure of how the data are clustered about the mean, is rather high, then each value differs from the mean (each value is distributed in a wide range around the mean).

## Discussion

Nanotechnology, perceived as one of the key technologies of this century, offers extraordinary perspectives for our everyday life (Cattaneo *et al*., 2010). The promise of this technology relies on the physical and chemical properties of nanoscale materials. The concern is that the unforeseeable toxicity may derive from the characteristics of these new fabricated nanomaterials, such as higher reactivity, unusual complex shapes and accessibility to cells (Bernardini *et al*., 2011).

Adequately assessing the biological effects of NPs is particularly difficult, as their *in vitro* and *in vivo* behaviours depend on numerous factors, including composition, size, shape, route of exposure or dosage. Additionally, unforeseen interferences affecting the outcome of a particular toxicological assay, inadequate controls, the wide range of biological assays, the different methods of quantifying dose, aggregation and dissolution are factors that contribute to confound the issue (Bernardini *et al*., 2011). The role of released metal ions also needs to be taken into account. In fact, there are recent *in vitro* studies showing that such particles can gradually dissolve in culture medium with the generation of ions (Papis *et al*., 2007b; Sabbioni *et al*., 2007). Therefore, the safety evaluation of nanomaterials cannot rely on the toxicological profile of the bulk material that has been historically determined. This has also led to the conclusion that the biological evaluation of NPs and/or products incorporating NPs should be performed on a case‐by‐case basis, making the risk assessment process of nanomaterials more complicated and slow. However, no experimental study has been dedicated to the possible anticipation of toxicological properties of NPs based on certain affinities in their chemical nature. With the aim to fill this gap, we have considered the case of zerovalent NPs derived from three transition metals of the same group (group VIIIb) of the periodic table such as Co‐, Fe‐ and NiNPs as well as of the corresponding ions potentially released in the biological environment. Grouping NPs according to similar physicochemical and toxicological properties could result in categories of NPs that integrate predicted and experimental data. We are indeed convinced that there is a need for a systematic approach to provide hazard identification data on the widest possible range of NPs. In the absence of such data, it is not possible to derive conclusions of the spectrum of toxicological effects that might be associated with such nanomaterials.

Based on the above‐mentioned considerations, we have conducted a series of experiments, using the SKOV‐3 and U87 cell lines, to gain insight on the modes by which different NPs may induce cytotoxicity. Our experiments have demonstrated that the effect on cell viability for the three NPs were significantly different and cytotoxicity ranking was CoNPs > NiNPs > FeNPs. As NP cytotoxicity could be ascribed to several factors such as dissolution in growth media during exposure, cell uptake, ions release within cells, stress stimuli related to surface, size and shape of the NP itself (Jiang *et al*., 2012; Lubick, 2008; Pisanic *et al*., 2013; Zhao *et al*., 2013), it is dutiful to examine most of these aspects. Consequently, we have, first, investigated by TEM the NP morphology, which also appeared different in shape and size within each NP. As dissolution depends, at least in part, on the pH and composition of the environment, in the preparation (e.g., sonication) and in the duration of exposure (Novak *et al*. 2013; Xie *et al*., 2012), we have also determined, by radiotracers and ICP‐MS methods, the amount of NPs dissolved in culture media. Our experimental evidence reported that for FeNPs and NiNPs this amount was quite low, while CoNPs released a consistent amount of Co^2+^. The two considered methodological approaches gave comparable but not similar results, consequently to establish a protocol to evaluate NP dissolution is extremely complex, and further studies to clarify this key point are needed. These findings, supported by those obtained from the metal uptake (Fig. 5), may explain the high toxicity of CoNPs even after short time exposures and suggest that, probably, the effect of CoNPs was mainly due to the ions rather than to the NP itself.

The course of normal cellular development as well as pathological changes are all believed to be driven by changes in gene expression. Similarly, modifications of the environment where cells and organisms live modulate gene expression profiles. It is, therefore, reasonable to think that also NPs might be capable of influencing gene expression. The study of these modifications can help to gain insights into the mechanisms of action of NP and, conversely, genes, whose expression is modified by exposure to NPs (Perconti *et al*., 2008), can be used as biomarkers (indicators of a biologic state) of exposure. In this study, we have taken into consideration the expression of the HSP70, MT2A, SDHB, MLL, CASP3, IQSEC1 and VMP1 transcripts.

CoNPs and NiNPs, and to a lesser extent ions, caused a marked increase of the expression of HSP70 of SKOV‐3. This is in agreement with the main function of HSP70 to protect cells from various injuries, such as elevated temperature, mechanical damage, hypoxia and reactive oxygen species (Silver and Noble, 2014). The response of U87 differs from that of SKOV‐3 and this will occur for several of the studied genes.

Metallothioneins are a family of highly conserved, cysteine‐rich, low molecular weight proteins capable of binding essential and non‐essential heavy metals through the thiol group of their cysteine residues. It is generally accepted that metallothioneins are involved in the detoxication of non‐essential metals and excess essential metals (Carpenè *et al*., 2007). The metallothionein gene family contains several members of which MT2A is most commonly expressed. In our experiments, MT2A was upregulated by Ni exposure (by NPs in SKOV‐3 and by ions in U87). Moreover, Ni is known to induce metallothionein expression in liver (Bauman *et al*., 1993) and lung (McDowell *et al*., 2000; Nemec *et al*., 2009). Furthermore, transgenic mice are resistant to Ni, while null mice are more susceptible (Wesselkamper *et al*., 2006).

SDHB is one of four protein subunits forming succinate dehydrogenase (SDH). The SDH complex is located on the inner membrane of the mitochondria and participates in both the citric acid cycle and respiratory chain. SDHB is assessed to check mitochondrial metabolism. SDHB has been found perturbed by some metal compounds (Bourdineaud *et al*., 2012; Monetti *et al*., 2002). We have not observed a clear response of this gene to our experimental conditions but for U87 cells exposed to Ni^2+^ where we have noticed a significant overexpression of this gene probably due to a mild oxidative stress.

MLL, whose mRNA was overexpressed in U87, after Co and Ni^2+^ exposure, is a histone H3K4 methyltransferase critical for normal hematopoietic development (Muntean and Hess, 2012). The MLL transcript has been shown to be responsive to several metals in BALB3T3 (Cinquetti *et al*., 2003).

Deregulation of CASP3, an executioner enzyme involved in apoptosis, underlies the need of controlled dismantling of intracellular components avoiding inflammation and damage to surrounding cells (McIlwain *et al*., 2013).

IQSEC1, also known as ARF‐GEP100, in addition to functioning as a guanine nucleotide exchange protein for ARF6 mediating internalization of beta‐1 integrin, is involved in signal transduction. It is a guanine nucleotide exchange factor that promotes binding of GTP to ADP ribosylation factor protein ARF6 and to a lesser extent ARF1 and ARF5 (Trinidad *et al*., 2010). ARF‐GEP100, through activation of ARF6, is therefore involved in the control of processes such as endocytosis of plasma membrane proteins, E‐cadherin recycling and actin cytoskeleton remodelling (Hiroi *et al*., 2006). ARF‐GEP100 appears particularly important in regulating cell adhesion; the reduction in the level of this protein causes enhanced spreading and attachment of cells (Dunphy *et al*., 2006). VMP1 is a putative transmembrane protein localized in the autophagosomal membrane, and associated with autophagy as well as cell adhesion, and membrane traffic. Cells deficient in VMP1 display defects in the context of the secretory pathway and in autophagosome biogenesis or maturation (Calvo‐Garrido *et al*., 2008). Notwithstanding their involvement in such important functions and preliminary indications on their responsiveness to metal exposure (Cinquetti *et al*., 2003; Papis *et al*., 2014), IQSEC1 and VMP1 were significantly induced only in U87 exposed to Ni^2+^.

In conclusion, real‐time PCR experiments aimed to evaluate modifications of the expression of genes involved in the cellular stress response (HSP70, MT2A), or susceptible to metal exposure (SDHB1, MLL, IQSEC1 and VMP1), or involved in specific cellular processes (CASP3), gave different response patterns in the two cell lines. This suggests that generalizing the induced cell response using a single cell line is not possible and strengthens the idea that the use of a combination of different *in vitro* models is unavoidable to perceive the toxicological risk due to NP exposure. Overall, this work underlines the difficulties in predicting NP toxicological properties on the only basis of their chemical characteristics. We, consequently, think that, at this stage of our knowledge, NP biological effects should be examined on a case‐by‐case basis after studies on different *in vitro* models. We realize that this approach might slow the risk assessment process of nanomaterials and we understand the need for a different strategy capable to reduce the necessity to resort to a case‐by‐case approach. The answer will be probably given by the adoption of *in silico* toxicology methods to create quantitative nanostructure–toxicity relationship models of the *in vitro* and *in vivo* effects of NPs (Winkler *et al*., 2011). Moreover, with the only exception of U87 exposed to Ni, our results suggest that metallic NPs have caused, on gene expression, similar effects to those caused by their corresponding ions.

## Conflict of interest

The authors report no conflicts of interest.
